# Blast phase of chronic myeloid leukemia presenting as early T‐cell precursor lymphoblastic leukemia

**DOI:** 10.1002/jha2.324

**Published:** 2021-10-26

**Authors:** Lianqun Qiu, Guilin Tang, Shaoying Li, Wei Wang, Sa A. Wang, Jie Xu

**Affiliations:** ^1^ Department of Hematopathology The University of Texas MD Anderson Cancer Center Houston Texas USA

**FIGURE 1:**
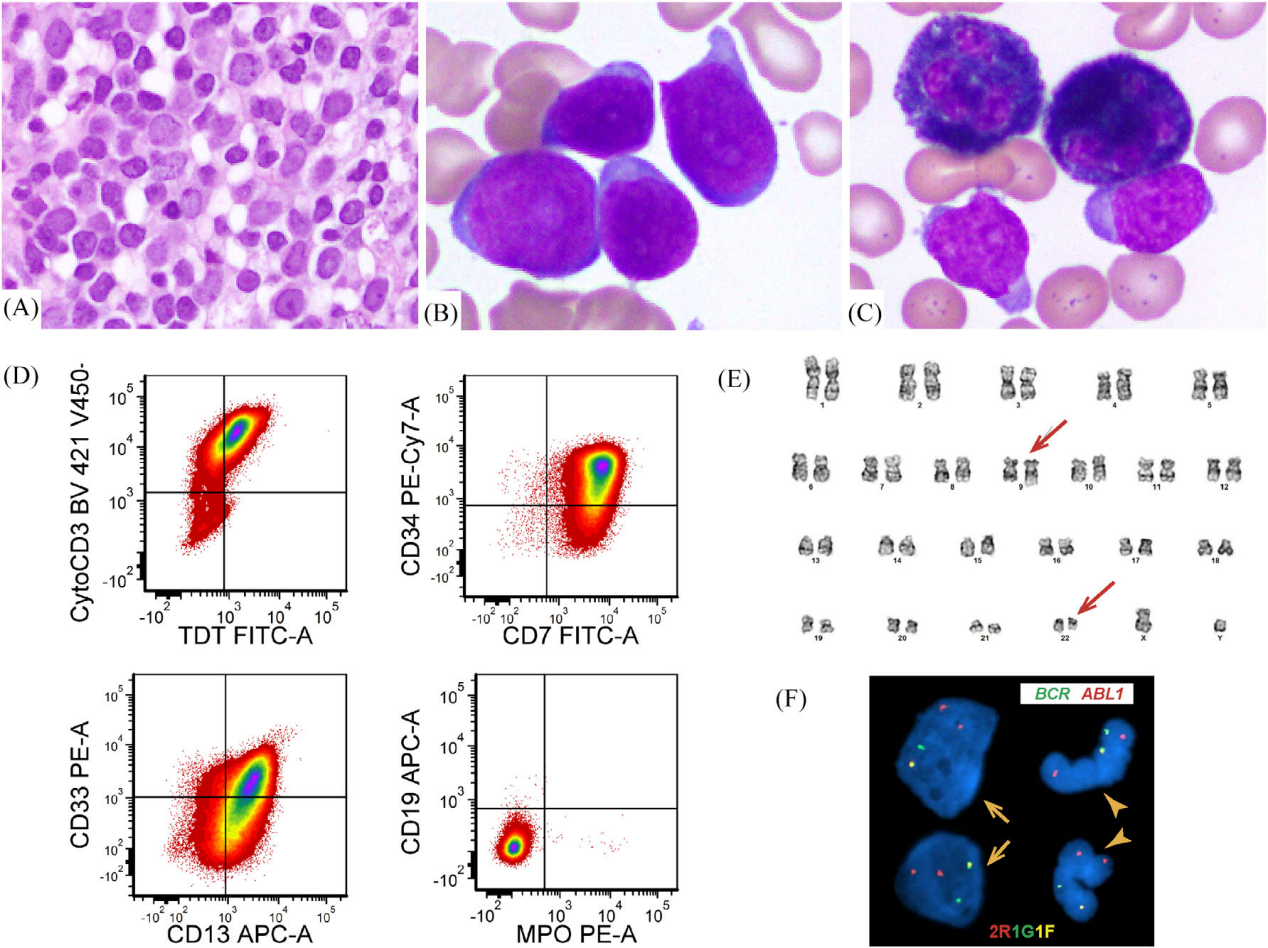
Morphologic, immunophenotypic, and cytogenetic findings of bone marrow

An 84‐year‐old man with an outside diagnosis of T lymphoblastic leukemia/lymphoma (T‐ALL) was transferred to our hospital. Complete blood count showed marked leukocytosis (325 × 10^9^/L) with 88% circulating blasts. The bone marrow biopsy showed hypercellular marrow with sheets of blasts (Figure 1A). The aspirate smears revealed small‐ to medium‐sized blasts (Figure 1B), which were negative for myeloperoxidase (MPO) by cytochemistry (Figure 1C). Flow cytometric immunophenotyping revealed T‐lymphoblasts: positive for CD3 (cytoplasmic), CD5 (< 75%), CD7, terminal deoxynucleotidyl transferase (TdT); negative for CD1a, CD2, CD3 (surface), CD4, CD8, and CD10. They also expressed multiple myeloid/stem cell markers, including CD13, CD33 (partial), CD34, CD117 (dim), HLA‐DR (partial), but negative for MPO and CD19, consistent with early T‐cell precursor (ETP)‐ALL (Figure 1D). Chromosomal analysis showed 46,XY,t(9;22)(q34;q11.2)[20] (Figure 1E), raising the concern for *de novo* Ph+ T‐ALL. However, fluorescence in situ hybridization (FISH) revealed *BCR‐ABL1* rearrangement (95% of cells) in both cells with round nuclei (blasts) and cells with segmented nuclei (neutrophils) (Figure 1F), supporting the diagnosis of chronic myeloid leukemia, blast phase (CML‐BP) rather than *de novo* Ph+ T‐ALL. The patient was diagnosed with CML‐BP with an early T‐cell precursor immunophenotype (CML‐BP‐ETP). He received one cycle of chemotherapy and tyrosine kinase inhibitor and died 40 days after diagnosis.

It can be challenging in distinguishing *de novo* Ph+ ALL from lymphoblastic CML‐BP, especially in patients with no known history of CML. Detection of *BCR‐ABL1* rearrangement in both round nuclei (blasts) and segmented nuclei (neutrophils) by FISH is critical in establishing the diagnosis of CML‐BP. CML‐BP‐ETP is very rare and reportedly associated with a very poor prognosis, therefore, it is important to recognize this type of CML‐BP and treat it appropriately.

